# Fingolimod effects in neuroinflammation: Regulation of astroglial glutamate transporters?

**DOI:** 10.1371/journal.pone.0171552

**Published:** 2017-03-08

**Authors:** De-Hyung Lee, Silvia Seubert, Konstantin Huhn, Lukas Brecht, Caroline Rötger, Anne Waschbisch, Johannes Schlachetzki, Alice Klausmeyer, Arthur Melms, Stefan Wiese, Jürgen Winkler, Ralf A. Linker

**Affiliations:** 1 Department of Neurology, Friedrich-Alexander-University, Erlangen-Nuremberg, Germany; 2 Department of Molecular Neurology, Friedrich-Alexander-University, Erlangen-Nuremberg, Germany; 3 Institute of Molecular Neurobiology, Ruhr-University, Bochum, Germany; Heinrich-Heine-Universitat Dusseldorf, GERMANY

## Abstract

Fingolimod is an oral sphingosine-1-phosphate-receptor modulator which reduces the recirculation of immune cells and may also directly target glial cells. Here we investigate effects of fingolimod on expression of astroglial glutamate transporters under pro-inflammatory conditions. In astrocyte cell culture, the addition of pro-inflammatory cytokines led to a significant downregulation of glutamate transporters glutamate transporter-1 (*slc1a2/*SLC1A2) and glutamate aspartate transporter (*slc1a3*/SLC1A3) expression on the mRNA or protein level. In this setting, the direct application of fingolimod-1 phosphate (F1P) on astrocytes did not change expression levels of *slc1a2 and slc1a3* mRNA. The analysis of both transporters on the protein level by Western Blot and immunocytochemistry did also not reveal any effect of F1P. On a functional level, the addition of conditioned supernatants from F1P treated astrocytes to neuronal cell culture did not result in increased neurite growth. In experimental autoimmune encephalomyelitis as a model of multiple sclerosis, fingolimod treatment reduced T cell and macrophages/microglia mediated inflammation and also diminished astrocyte activation. At the same time, fingolimod restored the reduced expression of *slc1a2* and *slc1a3* in the inflamed spinal cord on the mRNA level and of SLC1A2 and SLC1A3 on the protein level, presumably via indirect, anti-inflammatory mechanisms. These findings provide further evidence for a predominantly peripheral effect of the compound in neuroinflammation.

## Introduction

Multiple sclerosis (MS) is a chronic inflammatory disease mainly of young adults and presumably of autoimmune origin. On a histopathological level, central nervous system (CNS) lesions are characterized by T cell and macrophage infiltration, demyelination, axonal injury, and astrocyte activation associated with gliosis. The recent years witnessed a large body of new evidence on several immune cells types. Yet, much fewer insights on glial cells emerged at the same time, especially with regard to astrocytes. Astrocytes were long regarded as a purely passive cell type in the cascade leading to tissue damage in MS. However, more recent data point to an active role of astrocytes in the pathophysiology of the disease. Many of these insights stem from studies in animal models like myelin oligodendrocyte glycoprotein induced experimental autoimmune encephalomyelitis (MOG-EAE), which mimics many aspects of MS [1,2]. Among others, inflamed astrocytes in the local microenvironment around demyelinated lesions may actively participate in disease processes via production of cytokine, chemokines or growth factors, but also via regulating the local micromilieu of increased neurotransmitter levels which may act neurotoxic. Here, the regulation of glutamate metabolism is of special interest in the CNS. While glutamate acts as the main excitatory neurotransmitter, it may also lead to excitotoxicity and subsequently cell damage via increased intracellular calcium and an increase in reactive oxygen species if present at excess levels locally [3]. Specifically, astrocytes may play an important role in glutamate homeostasis of the CNS via expression of several glutamate transporters like glutamate transporter-1 (SLC1A2) or glutamate aspartate transporter (SLC1A3) on astrocytic processes near synapses thereby enabling astrocytes for glutamate uptake and controlled release for neurotransmission [4]. Importantly, both in MS and EAE lesions, the expression of glutamate transporters is reduced while glutamate receptors on neurons are increased, indirectly indicating the presence of excitotoxic mechanisms of tissue damage during neuroinflammation [5–7].

Fingolimod is a new orally available compound for the treatment of relapsing-remitting MS, which has recently been licensed in many countries worldwide. Fingolimod acts via modulation of sphingosin-1 phosphate receptors (S1P), which are expressed on many cell types throughout the body. While, in MS, it is primarily thought to act via inhibiting the egress of pathogenic lymphocytes from the lymph node [8], the presence of S1P subtypes on glial cells and neurons—together with the capacity of fingolimod to cross the blood brain barrier [9]—opens the avenue for additional direct effects in the CNS. In the CNS, several S1P subtypes are found. On neurons, mostly S1P1 are present while oligodendrocytes are characterized by a predominant expression of S1P5. In these CNS cell types, fingolimod may protect from apoptosis and induce expression of neurotrophic factors like brain derived neurotrophic factor, which plays an important role for tissue protection during autoimmune neuroinflammation [10–12].

In addition, several lines of evidence point to a possible role of astrocytes as an important fingolimod target in the CNS. In MS lesions, astrocytes express S1P subtypes S1P1 and, to a lesser degree also S1P3 [13]. Binding of sphingosin-1 phosphate to its receptors on astrocytes was shown to induce astroglial activation [14]. Calcium imaging studies in mixed cultures from embryonic rat cortex revealed that astrocytes are the major cell type responsive to fingolimod. At the same time, the application of fingolimod-1 phosphate (F1P) on astrocyte-enriched cultures increased astrocyte migration [15]. Importantly a conditional knockout of S1P1 on astrocytes resulted in a milder course of EAE. Along the same line, only astroglial S1P1 were shown to be required as the pivotal CNS S1P for the fingolimod mechanism of action in EAE [16]. However, to date, there is little evidence on the direct cellular effects of fingolimod on astrocytes in neuroinflammation. Recently, a combination of *in vivo* and vitro studies suggested that fingolimod may reduce astrocyte-mediated neurodegeneration via inhibition of astroglial nitric oxide production [17]. Since blockade of astroglial S1P3 reduces neuronal glutamate release [18], we hypothesized that S1P modulation may beneficially affect the expression of astroglial glutamate transporters SLC1A2 and SLC1A3 during autoimmune neuroinflammation.

Here we thus investigate effects of fingolimod on astroglial glutamate transporters under pro-inflammatory conditions *in vitro* and *ex vivo*.

## Materials and methods

### Mice, EAE induction, and fingolimod treatment

Mice were initially purchased from Charles River (Sulzfeld, Germany) and further bred in-house on the C57BL/6 background for at least 10 generations. All mice were housed at the in-house animal care facility of the University of Erlangen under a 12-h our day-night-cycle and standardized environment.

For induction of EAE, male and female mice 8–11 weeks of age were anaesthetized (ketamine/xylazine 80 mg per kg/8 mg per kg) and received a total of 200 µg MOG_35-55_ and 200 µg Freund´s complete adjuvant (CFA), containing 4 mg/ml *M*. *tuberculosis* (H37RA) administered by two subcutaneous injections of 50 µl emulsion left and right to the tail base. Pertussis toxin (200 ng/mouse) was applied intraperitoneally on days 0 and 2 post induction. Clinical evaluation was performed on a daily basis by a 10-point scale ranging from 0, normal; 1, limp tail tip, 2, limp tail 3, impaired righting; 4, gait ataxia; 5, paresis of one hindlimb, 6, incomplete paraparesis of both hindlimbs; 7, complete paraparesis, 8, tetraparesis; 9 moribund, 10, death. Mice were sacrificed if reaching a disease score of 7. To achieve optimal CNS effects, mice received fingolimod at a dosage of 3 mg/kg or carrier alone (consisting of an 1:1 solution of ethanol:PBS, pH 7.2) as sham treatment via oral gavage once daily. All experiments were in accordance with the German laws for animal protection, and were approved by the local ethic committees of the University of Erlangen (AZ 54–2532.1-56/12).

### Immunohistochemistry and stereological quantification

EAE mice were perfused with 4% PFA (paraformaldehyde, Sigma) and the spinal cord and spleen were removed and embedded in paraffin before sectioning in 5 μm slices. Luxol Fast Blue staining was performed for evaluation of demyelination and Bielschowksy silver impregnation for axonal integrity/damage. Immunohistochemistry was performed on 5 μm thick paraffin sections (αCD3 1:200; Serotec; Wiesbaden, Germany; αMac-3 1:200; BD Pharmingen; Heidelberg, Germany; αGFAP 1:1000; DAKO; Hamburg, Germany; αAPP 1:1000; Millipore). Quantification of axonal densities, cellular infiltrates, and the degree of demyelination were performed by a blinded observer on nine independent spinal cord cross sections per mouse. Cellular infiltrates were quantified by overlaying a stereological grid onto sections and counting infiltates per mm^2^ white matter. Demyelinated areas were semi-automatically assessed by CellD Software (Olympus, Hamburg, Germany). Nine visual fields of the cervical, thoracic and lumbar spinal cord were used for quantification of axonal preservation counted on a 100 mm diameter grid as described previously [11].

Double labelling of SLC1A2 (1:500 rabbit, Thermo Fisher Scientific; Darmstadt, Germany) and SLC1A3 (1:500 rabbit, Abcam; Cambridge, UK) with GFAP (1:1000 mouse, Biolegend; Fell, Germany) was visualized using laser scanning microscopy (Leica Microsystems, Wetzlar, Germany).

### Real-time PCR

Gene expression was analysed by real time PCR. Cultured astrocytes were lysed in RLT-buffer (RNeasy kit, QIAGEN, Hilden, Germany) and total RNA was isolated using the RNeasy kit following the manufacturer’s instructions (QIAGEN). RNA yield was quantified by absorbance measurements at 260 nm. 50–500 ng of total RNA were used per reaction to reversely transcribe RNA into cDNA, using QuantiTect transcriptase according to the protocols (QIAGEN). PCR reactions were performed at a 5 µl scale on a qTower real time PCR System (Analytic Jena) in triplicates. Relative quantification was performed by the ΔΔCT method, normalizing target gene expression either on *Actb*/β-Actin or Rn18s as housekeeping genes. Custom made primers were used to amplify murine slc1a3 mRNA (Thermo Fisher Cat. # 4331182) or murine slc1a2 mRNA (Thermo Fisher Cat # 4331182).

### Astrocyte culture

Astrocytes were isolated from murine primary mixed glial cell cultures, which were prepared from pups P1-3 as described previously [5]. To control for purity of the preparation, glial fibrillary acidic protein (GFAP) staining (1:100, Dianova, Hamburg, Germany) was performed as described earlier. Astrocyte cultures were found to be of >98% purity. After being reseeded, astrocytes were kept in culture for 12 days before stimulation with tumor necrosis factors alpha (TNFα 100 U/ml) and interleukin 1 beta (IL-1β 10 ng/ml). Fingolimod-1 phosphate (F1P) was obtained from Novartis, Basle, Switzerland, dissolved in chloroform at a concentration of 0.5 mg/ml as stock solution and was added to the cultures together with cytokines at a final concentration of 100 nM. Control cultures were treated chloroform only diluted in water resulting in a final concentration of 0.7 pM.

### PC 12 cell based neurite growth assay

In this assay, a TrkB overexpressing PC12 neuronal cell line was employed. Cells were plated in poly L-ornithine and subsequently laminine covered 24 well plates and cultured at a density of 20.000 cells/well in 500 µl of DMEM medium with the addition of 5% fetal calf serum, 10% horse serum and 1% penicillin/streptomycin as well as G418 as antibiotic at 4.4% CO_2_ and 37°C. After a day of culture, astrocyte supernantants were added in duplicates at a concentration of 1:10; treatment with BDNF at 10 ng/ml served as positive control. After two days of culture and paraformaldehde fixation as well as hematoxylin-eosin staining, neurite lengths were counted in a blinded manner under a microscope with 100 fold magnification. Per preparation, 100 cells were analysed summarizing neurite lengths per individual cell with 5 µm as minimal cutoff.

### Western blotting

Cultured astrocytes were harvested in 1xRipa lysis buffer [10xRipa: 150 mM NaCl, 38.5mM SDS, 50mM Tris, 134mM SDOX, 0.5 mM EDTA, 1% NP40, complete protease inhibitor cocktail Complete Mini and phosphatase inhibitor cocktail PhosStop (Roche Diagnostic GmbH, Mannheim, Germany)] and centrifuged at 10,000 rpm for 10 min. Protein concentration was determined with BC Assay Protein Quantitation Kit (Interchim) and equal amounts of proteins were analyzed by Western blotting. SLC1A2 protein was detected by using rabbit anti-SLC1A2 (Thermo Fisher Scientific, 1:500) and SLC1A3 by using rabbit anti SLC1A3 (Abcam; 1:300). Mouse anti-β-actin (1:1000, Millipore clone C4) or anti-glyceraldehyde 3-phosphate dehydrogenase (GAPDH) was blotted as loading control. All blots were at least performed three times. Signal capture was conducted using a Fusion FX7 detection system (PeqLab). Densitometric quantifications were performed using the Bio1D software (Vilber Lourmat) by normalizing signals to beta actin or GAPDH expression (for further details on blotting and quantification see also [19]).

### Statistical analysis

Statistical analysis was performed using GraphPad Prism (GraphPad Software Inc., La Jolla, CA). Data from two groups were analyzed by unpaired t-test or Wilcoxon rank sum test after checking for normal distribution for *ex vivo* and *in vitro* data and by Mann-Whitney U test for EAE data. For three or more groups, a Kruskal Wallis test was employed. Data are presented as mean ± SD or mean ± SEM. *p<0.05, **p<0.01, or ***p<0.001 were considered to be statistically significant.

## Results

### Fingolimod increases slc1a2 expression in inflamed astrocye culture on the mRNA, but not on the protein level

**Fig 1:**
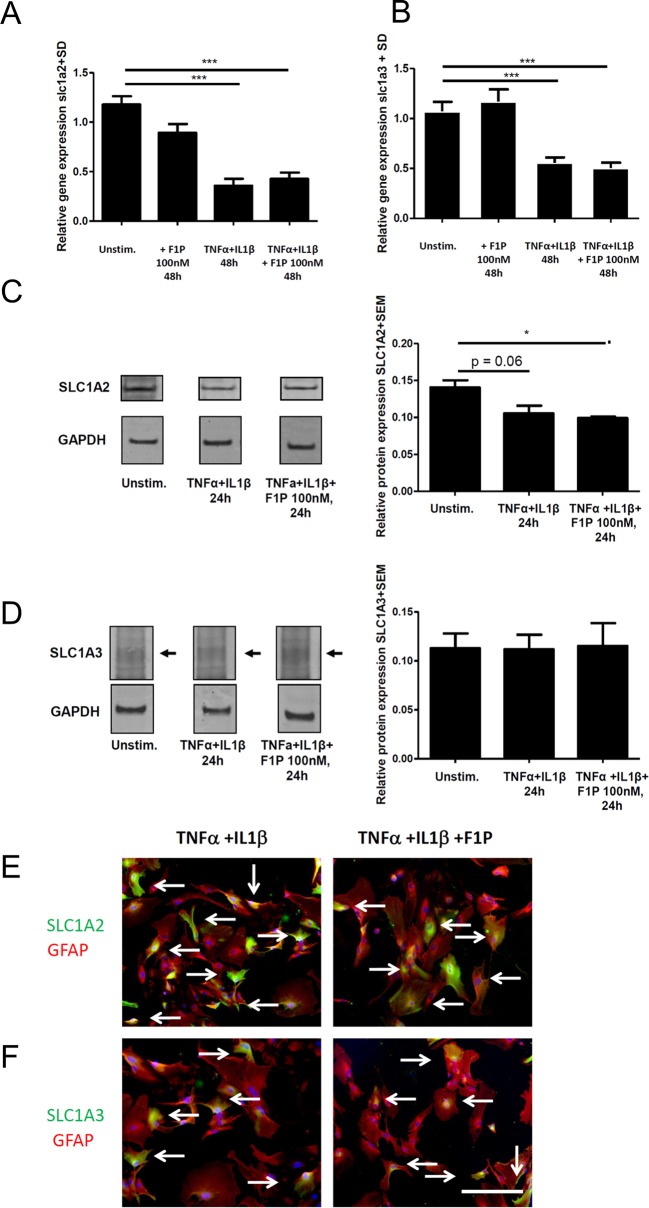
Effects of fingolimod-1 phosphate on glutamate transporter mRNA and protein levels in astrocyte cell culture. RT-PCR analysis of (A) *slc1a2* expression or (B) *slc1a3* expression after astrocyte culture for 12 days and 48 hours of stimulation with TNF-α (100 U/ml) and IL-1β (10 ng/ml) versus naïve controls and with our without addition of F1P at 100 nM, n = 17/3/9/14 per group. (C,D) Western Blot analysis of (C) SLC1A2 protein expression or (D) SLC1A3 protein expression in astrocytes cultured for 12 days either as naïve cells or after stimulation with 100 U/ml TNF-α and 10 ng/ml IL-1β for 48 hours with or without F1P at 100 nM. Densitometric analysis of SLC1A2 or SLC1A3 protein levels was performed in relation to glyceraldehyde 3-phosphate dehydrogenase (GAPDH) as loading control, n = 3–4 per group, data are pooled from two experiments. Arrows indicate specific band. (E,F) Confocal imaging after immuncytochemistry for GFAP (red) and SLC1A2 or SLC1A3 (green) in inflamed astrocyte culture (stimulation with TNF-α and IL-1β) with or without addition of 100nM F1P for 48 hours. Images from representative cultures are shown. Bar = 20 µm for G,H. Data are given as mean ± SD for RT-PCR data or mean ± SEM for Western Blotting. * p < 0.05 or *** p < 0.01, t-test or Kruskal Wallis-test.

To dissect actions of fingolimod on astroglial glutamate transporters, we first performed studies in astrocyte culture in naïve cells and under pro-inflammatory conditions. Stimulation of naive or F1P treated astrocytes with IL-1β and TNFα resulted in a significant reduction of both, *slc1a2* and *slc1a3* expression on the mRNA level (Fig 1A and 1B). In naïve as well as stimulated astrocytes, the application of F1P at 100 nM did not result in direct effects of *slc1a2* or of *slc1a3* mRNA expression versus sham treated controls (Fig 1A and 1B).

To confirm these data on the protein level, we performed Western Blot experiments for SLC1A2 and SLC1A3 with cellular extracts from astrocytes after 12 days of culture and included a densitometric analysis in relation to GAPDH as housekeeping gene. Well in line with the gene expression data, stimulation with IL-1β and TNFα led to decreased protein levels of SLC1A2 (Fig 1C). Yet, there was no significant difference in SLC1A2 or SLC1A3 protein levels when comparing stimulation of astrocytes with IL-1β and TNFα to inflamed conditions with the addition of 100 nM F1P after 48 hours (Fig 1C and 1D). Immuncytochemistry for GFAP and SLC1A2 (Fig 1E) or SLC1A3 (Fig 1F) with confocal imaging also did not detect any difference in glutamate transporter expression between inflamed astrocytes with or without addition of F1P.

### Fingolimod exerts beneficial effects on the clinical course as well as parameters of inflammation and degeneration upon treatment initiation at different stages of EAE

**Fig 2:**
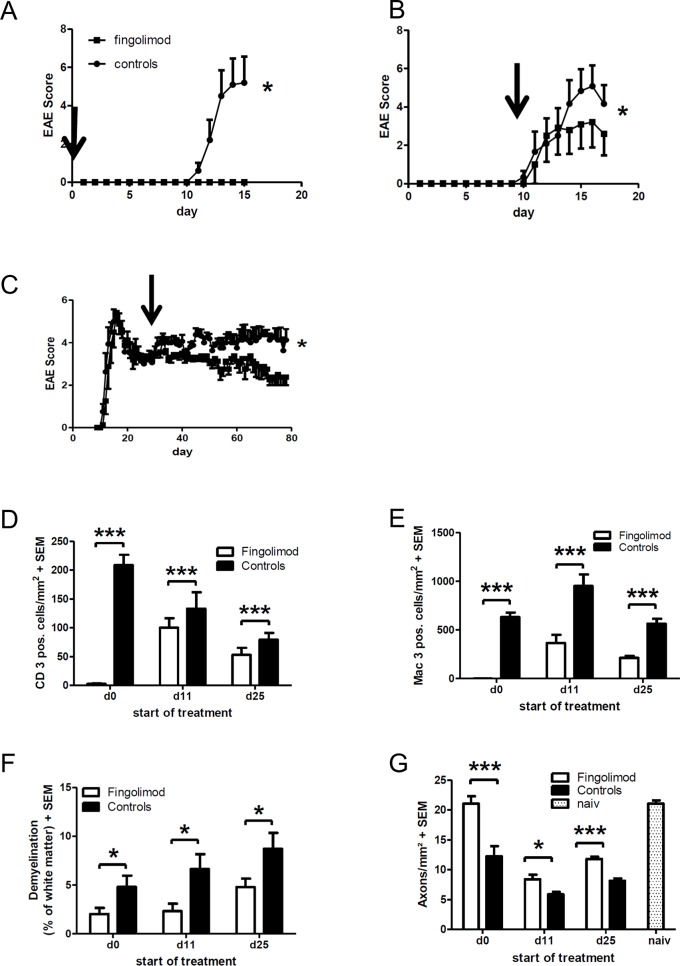
Clinical course and blinded histpathological analysis of inflammation and degeneration after treatment of MOG-EAE with fingolimod at 3 mg/kg/d. (A) Clinical courseo f EAE after prophylactic treatment starting from day 0 after immunization (n = 6 per group). (B) Treatment initiation at the beginning of disease (day 11 p.i., n = 6 per group). (C) Treatment initiation at the early chronic phase of the disease (day 25 p.i., n = 8 per group). (D-G) Blinded histopathological quantification of spinal cord cross sections after staining for (D) CD3 positive T cells, (E) Mac-3 positive macrophages and microglia, (F) demyelination with the Luxol Fast Blue technique, and (G) axonal densities with Bielschowsky silver impregnation. In A-C, the day on the X axis indicates the start of treatment: directly after immunization (d0, n = 6 per group), at the first sign of symptoms (d11, n = 6 per group) or at the early chronic phase of the disease (d25, n = 8 per group). Experiments were analysed at the maximum of disease (days 15 or 17 p.i., respectively) for treatment start on day 0 and 11 p.i. and in the later chronic phase of EAE (day 80 p.i.) after treatment start on day 25 p.i. All data are given as mean ± SEM. Arrows indicate start of treatment.* p < 0.05, *** p < 0.001, Mann-Whitney test.

We next focused on the analysis of fingolimod effects in the spinal cord of mice suffering from EAE. To this end, mice suffering from EAE were fed with 3 mg/kg fingolimod per day via oral gavage. Treatment was started either prophylactically (day 0 p.i.) or in a therapeutic approach beginning at the onset of disease (day 11 p.i.) or at the early chronic disease phase (day 25 p.i.). Mice were then followed until the first maximum of disease (days 15–17 p.i.) or—in the case of late therapy start—until the very late phase of disease (day 80 p.i.). In the prophylactic setting, fingolimod treatment completely prevented the development of EAE symptoms as compared to sham treated controls (Fig 2A). Upon treatment start at the onset of disease, fingolimod displayed a fast onset of efficacy and significantly ameliorated the course of EAE until the first maximum of the disease at day 15–17 p.i. (Fig 2B). After initiation of therapy on day 25 p.i., there was a prolonged onset of efficacy with a lag of about 10 days. Afterwards, fingolimod reduced the severity of EAE to a similar degree as with early treatment start during disease onset with sustained effects until the late course of the disease (Fig 2C).

In the next step, we performed a histopathological analysis of spinal cord cross sections comprising cervical, thoracic and lumbar parts. In the preventive as well as in both early and late therapeutic treatment paradigms, fingolimod significantly reduced infiltrating T cells as well as microglia and macrophages in the spinal cord (S1A–S1D Fig). Consistently, fingolimod treatment reduced demyelination and led to preservation of axonal densities in the spinal cord at all time points (S1E–S1H Fig).

### Fingolimod reduces astrocyte activation and increases astroglial expression of glutamate transporters in EAE on the mRNA level

**Fig 3:**
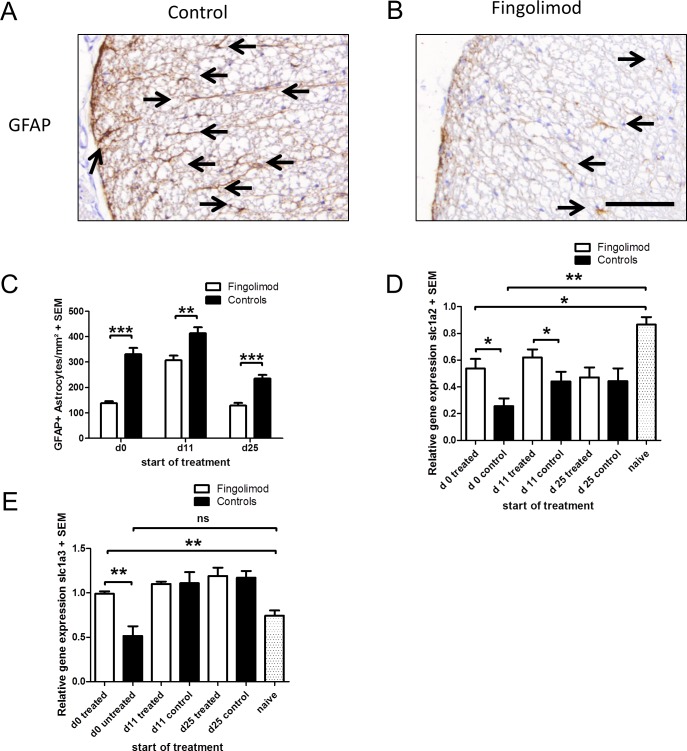
Effects of fingolimod on astrocyte activation and glutamate transporter mRNA levels in the spinal cord during EAE. (A, B) Representative GFAP staining of spinal cord cross sections from a mouse treated with fingolimod in a preventive setting and a sham treated control at the maximum of EAE. Note the reduced GFAP staining after fingolimod treatment. Bar = 100µm (C) Blinded quantification of GFAP immunreactivity as marker of astrocyte activation on spinal cord cross sections. (D) RT-PCR analysis of *slc1a2* expression. (E) RT-PCR analysis of s*lc1a3* expression, n = 6–8 per group. The mRNA expression of a prophylactically treated mouse is set to 1 as reference. All data are given as mean ± SEM. Please note that data are compiled from separate experiments with different starting points of fingolimod application (d0, 11 or 25). The day (d) on the X axis indicates the start of treatment directly after immunization (d0, n = 6 mice per group), at the first sign of symptoms (d11, n = 6 mice per group) or at the early chronic phase of the disease (d25, n = 8 mice per group). Experiments were analysed at the maximum of disease (days 15 or 17 p.i., respectively) for treatment start on day 0 and 11 p.i. and in the later chronic phase of EAE (day 80 p.i.) after treatment start on day 25 p.i. * p < 0.05, ** p < 0.01; *** p < 0.001, Mann Whitney test.

Blinded quantification of activated astrocytes after GFAP staining revealed a significant reduction of GFAP expression in the analysis of the preventive as well as both therapeutic treatment paradigms (Fig 3A–3C). In RT-PCR analyses, we subsequently investigated the mRNA expression of spinal cord glutamate transporters *slc1a2* und *slc1a3*. As compared to naive mice, induction of EAE led to a significant down-regulation particularly of *slc1a2*. This down-regulation of *slc1a2* was significantly preserved after preventive treatment as well as early therapeutic application of fingolimod in EAE (Fig 3D). In contrast, expression of *slc1a3* was significantly higher only after preventive fingolimod application (Fig 3E).

### Fingolimod does not exert effects on glutamate transporters in EAE on the protein level

**Fig 4:**
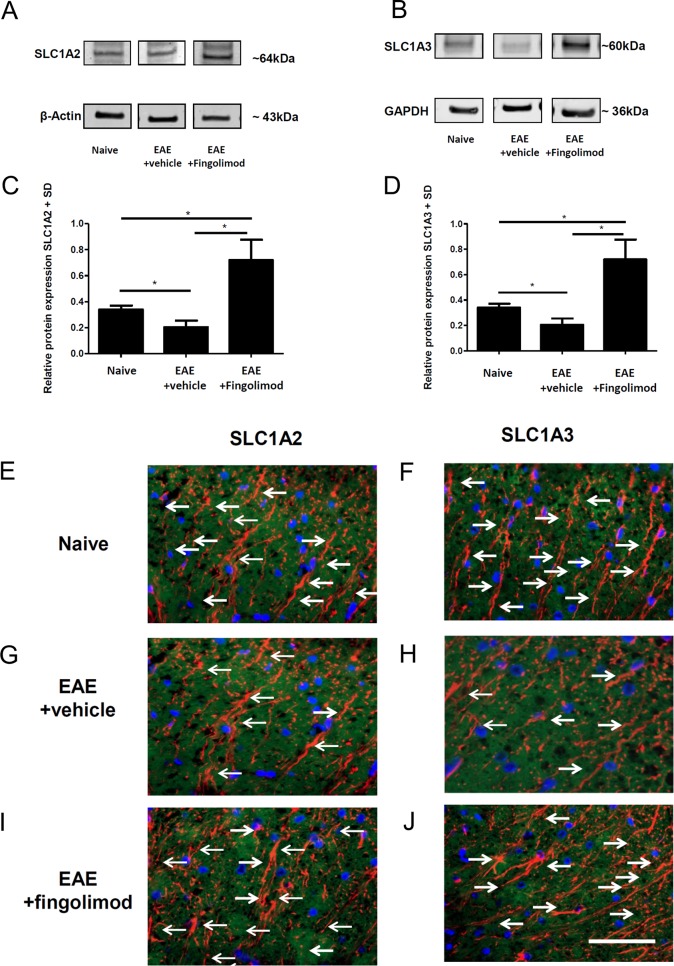
No effects of fingolimod on spinal cord glutamate transporter protein levels during EAE. (A-D) Western Blot analyses of spinal cord homogenates relative to GAPDH (A) or beta actin (C) with the respective densitometry relative to the housekeeping gene (B,D). At the maximum of EAE, there was a decrease for SLC1A2 (A,B) and SLC1A3 protein levels (C,D) as compared to naïve mice which was restored after fingolimod treatment (3 mg/kg once daily). One out of three experiments is shown, n = 3 per group. (E-H) Laser scanning microscopy of spinal cord cross sections after staining for GFAP (red) and SLC1A2 (green, E,F) or SLC1A3 (green, G,H). Arrows indicate double labelled profiles. Representative images of spinal cord cross sections are shown. Bar = 50 μm.

To further investigate the regulation of astroglial glutamate transporters on the protein level, we performed Western Blot analyses as well as immunohistochemical studies for SLC1A2 or SLC1A3 with spinal cord specimen obtained at the maximum of disease after preventive fingolimod application. Western Blot analyses with densitometric analyses in relation to GAPDH as housekeeping gene revealed a significant decrease in SLC1A2 expression at the maximum of EAE as compared to controls. This decrease was restored upon fingolimod treatment (Fig 4A and 4B). Similar findings were made upon analysis of SLC1A3 protein levels with densitometry in relation to beta actin (Fig 4C and 4D). Finally, we performed confocal laser scanning microscopy after double staining for GFAP and SLC1A2 or SLC1A3 on spinal cord cross sections (Fig 4E–4H). After staining for GFAP and SLC1A2 and SLC1A3, we found a decrease in double labeled astrocytes in EAE mice as compared to naïve controls. In contrast, fingolimod treatment restored expression to levels of naïve controls for both transporters (Fig 4I and 4J).

### Fingolimod conditioned astrocyte supernatants do not exert growth promoting effects in a neuronal cell line

**Fig 5:**
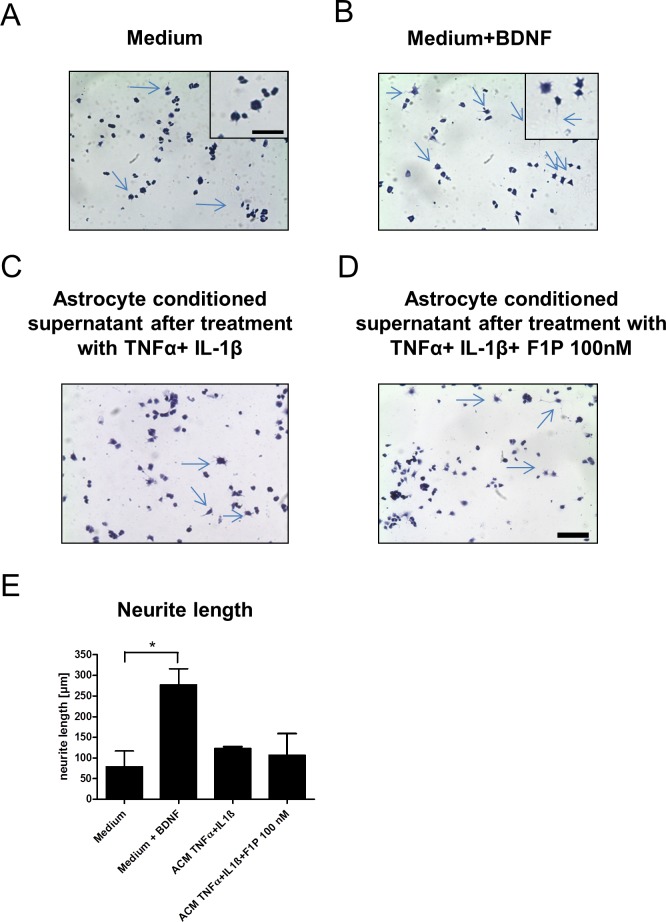
Fingolimod conditioned astrocyte supernatants do not exert growth promoting effects in PC 12 cells. (A-D) Representative images of PC12 cells in culture after hematoxylin eosin staining. Insets in (A) and (B) show higher manigifcation with representative neurite growth indicated by arrows. Bar indicates 50 µm in D and 20 µm in inset. As compared to (A) medium only as negative control and (B) the addition of BDNF as positive control, the addition of conditioned supernatnats from IL-1β and TNFα inflamed astrocytes with or without F1P treatment at 100 nM (C,D) did not lead to increased neurite lenght. (E) Blinded quantification of neurite lenghts in PC 12 cell culture. ACM, astrocyte conditioned supernatant. Data are given as mean ± SEM, n = 3 per group, 1 out of 2 experiments is shown. * p < 0.05 for medium versus addition of BDNF as positive control, Kruskal Wallis test.

To analyze the functional relevance of F1P treatment on astrocytes including possible effects of glutamate excitotoxicity on neurites, supernatants from F1P treated IL-1β and TNFα inflamed astrocytes were tested in a PC12 cell based neuronal growth assay. In comparison to medium as negative control and the addition of BDNF as positive control, neither supernatants from inflamed astrocyte nor those with addition of 100nM F1P significantly supported neurite growth (see representative images in Fig 5A–5D). Similar effects were seen when testing supernatants of astrocyes without pro-inflammatory cytokine stimulation, again without differences between cultures treated with 100 nM F1P and controls (images not shown). The blinded quantification of neurite lenghts in culture is shown in Fig 5E.

## Discussion

In the present study, we show beneficial effects of fingolimod on inflammation and astrocyte activation in the model of EAE with effects on astroglial glutamate transporters on the mRNA, but not on the protein level.

The beneficial effects of fingolimod on the course of EAE extend previous reports on the efficacy in acute and relapsing rodent animal models of MS [20–22]; as well as chronic MOG-EAE models in DA rats and C57BL/6 mice [23,24]. While these studies followed mice until the early chronic disease phase at the latest, our study provides a long-term observation of the clinical course in the MOG-EAE model. Similar to studies in DA rats [25], fingolimod was not only effective upon preventive application and treatment early after onset of disease, but a similar efficacy was seen upon start of therapy not earlier than the early chronic phase of the disease. While many compounds with very different modes of action are effective in EAE, particularly if given preventively, only few approaches show consistent effects upon treatment initiation in the later phases of the disease.

At all time-points of treatment initiation, fingolimod therapy led to beneficial effects on demyelination, axon densities, and astrocyte activation. Together with the presence of S1P receptors on neurons and glial cells and the capacity of fingolimod to cross the blood brain barrier [9], these positive actions on CNS cells open the possibility of direct CNS effects of fingolimod. This concept is further underscored by the efficacy of fingolimod in stroke models (for overview see [26]) and in an animal model of Rett syndrome [10]. Further adding to this notion, several studies in cell culture showed effects of fingolimod on astroglial cells [16,17] as well as on oligodendroglial proliferation and differentiation [27]. Yet, at all time points of treatment in EAE, fingolimod also significantly reduced numbers of infiltrating T cells and macrophages/microglia in the spinal cord, even after late treatment initiation in the early chronic phase of the disease. Thus, all positive effects on neurons and glial cell may also—at least in part—be due to a reduction of inflammatory damage to the CNS which renders direct CNS effects of fingolimod hard to delineate in EAE, but also in models of stroke and intracerebral hemorrhage [28,29].

While many studies in EAE and also in experimental stroke models point at direct effects of fingolimod in the CNS via astrocytes [16,30], only few approaches investigated the mechanism by which fingolimod may act on this cell type [5]. Astrocytes play an important role in the regulation of glutamate metabolism and thus the delimitation of glutamate excitotoxicity [5]. Here, astroglial glutamate transporters are of particular interest. Previous work shows a downregulation of astroglial glutamate transporters under pro-inflammatory conditions [31,32], as also corroborated in the present study.

Glutamate excitotoxicity may have an important role for neuro-axonal damage in MS and EAE lesions [33]. Besides putative direct effects on astroglial gluatamte transporters, fingolimod may also indirectly affect glutamate transporters via potentiating 14-3-3 phosphorylation [34]. These effects on 14-3-3 may inhibit protein kinase C which is known to regulate glutamate transporters under pro-inflammatory conditions [35]. However, the lacking direct effect of fingolimod on astroglial glutamate transporter protein levels *in vitro* does not argue for a definite contribution of such a direct mechanism to the *in vivo* effect in neuroinflammation. This is also underscored by the negative effect in a neuronal growth assay. Rather, the anti-inflammatory effect of fingolimod may result in a restoration of the reduced expression of SLC1A2 and SLC1A3 in EAE on the mRNA and protein level as compared to naïve controls.

In addition to some data on the application of fingolimod in the very late chronic disease phase of a relapsing-remitting EAE model [36], our data may help to inform about molecular reasons for the negative results from the INFORMS trial investigating the efficacy of fingolimod in primary progressive MS [37]. In MS research, further studies on direct effects of S1P modulation in the CNS as well as on other compounds directly acting on CNS cells will be awaited with great interest.

## Supporting information

S1 Fig Representative images of spinal cord cross sections. (A,B) After staining for CD3 (T cells), (C,D) Mac-3 (macrophages/microglia), (E,F) demyelination (Luxol Fast Blue), (G,H) activated astrocytes (GFAP), and (I,J) axons (Bielschowsky silver impregnation). Controls are shown on the left; fingolimod treatment is displayed on the right side of the panel. Bar 500 µµm in E,F and 100 µm for all others. Arrows denote demyelinated lesions or labelled cells.
(TIF)
