# Supplementary figures and images for: Fingolimod effects in neuroinflammation: Regulation of astroglial glutamate transporters?
Source: PLoS One. 2017 Mar 8;12(3):e0171552. doi: 10.1371/journal.pone.0171552 (PMC5342171; doi:10.1371/journal.pone.0171552)

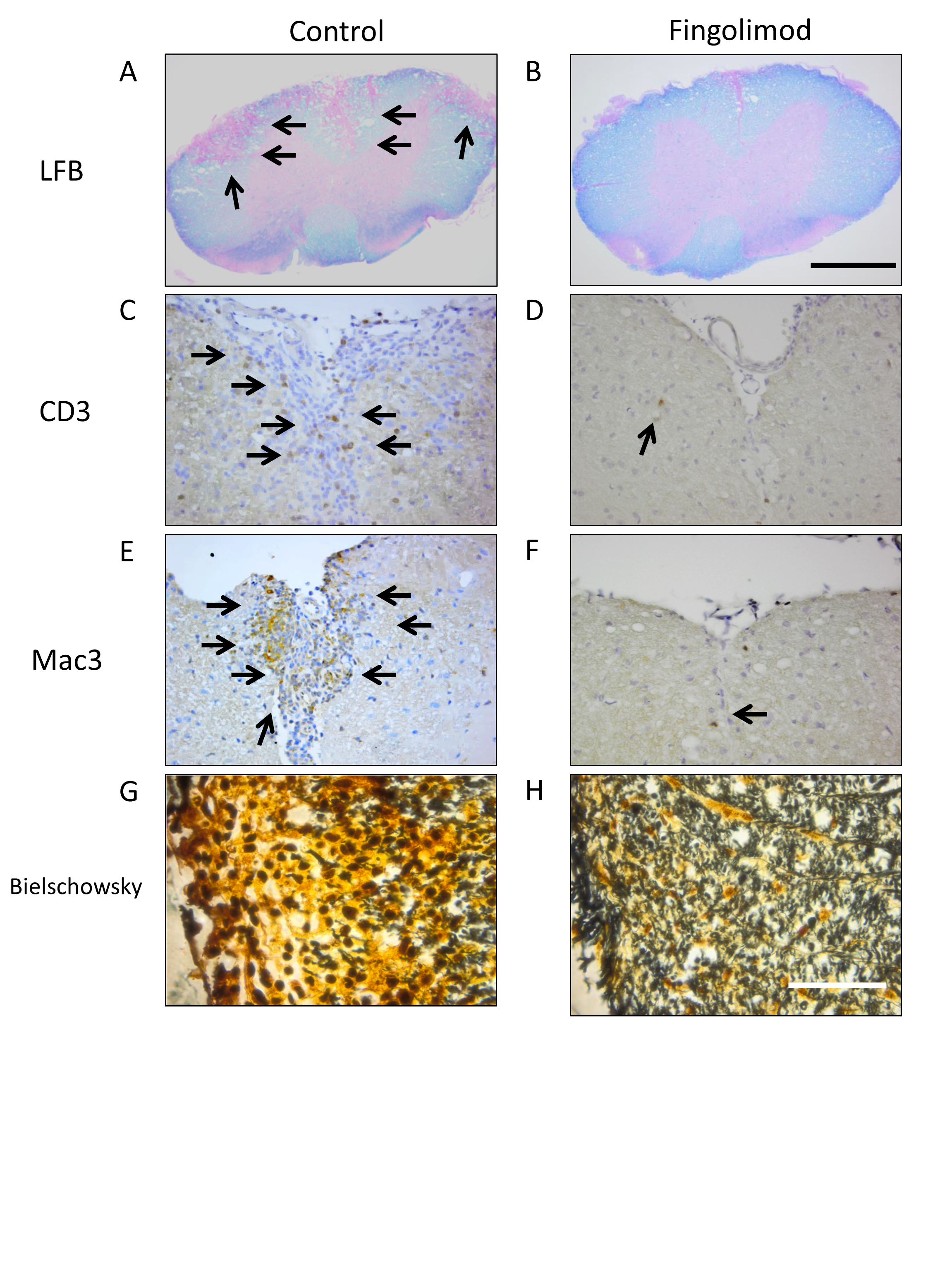

Supplement: S1 Fig — (A,B) After staining for CD3 (T cells), (C,D) Mac-3 (macrophages/microglia), (E,F) demyelination (Luxol Fast Blue), (G,H) activated astrocytes (GFAP), and (I,J) axons (Bielschowsky silver impregnation). Controls are shown on the left; fingolimod treatment is displayed on the right side of the panel. Bar 500 µµm in E,F and 100 µm for all others. Arrows denote demyelinated lesions or labelled cells. (TIF) [file pone.0171552.s001.TIF]
